# User Experiences of Prescription and Over-The-Counter Drug Abuse in Aden City, Yemen

**DOI:** 10.3390/pharmacy6030099

**Published:** 2018-09-13

**Authors:** Ebtesam A. Abood, Jenny Scott, Mayyada Wazaify

**Affiliations:** 1 Department of Clinical Pharmacy, Faculty of Pharmacy, Lebanese International University (LIU), Aden, Yemen; ph.ebtisam@gmail.com; 2 Department of Biopharmaceutics and Clinical Pharmacy, School of Pharmacy, The University of Jordan (JU), Amman 11942, Jordan; 3 Department of Pharmacy and Pharmacology, University of Bath, Bath BA2 7AY, UK; j.a.scott@bath.ac.uk

**Keywords:** community pharmacy, drug misuse, OTC drugs, non-prescription, prescription, qualitative experience, Yemen, violence

## Abstract

Khat chewing is commonplace in Yemen, but little else is known about the misuse of other drugs, especially how such misuse may intersect with Khat use. The aim of this study was to investigate misuse of prescription and over-the-counter (OTC) drugs in community pharmacies in Aden city, from the users’ perspective. A qualitative in-depth-interview study was undertaken with fifteen known or suspected drug misusers, recruited through community pharmacies. Thematic analysis was used to identify the main emergent themes around experience of prescription and OTC drug misuse. The majority of interviewees were male (*n* = 11/15) with an age range of 21–40 years. Benzodiazepines, Tramadol, and Ketoprofen were the most commonly misused drugs. Four main themes were identified: Experience sought with drugs; awareness of problematic drug use; pattern and methods of misuse; and the role of healthcare professionals in responding to misuse. The study highlighted different issues, such as the practice of mixing different OTC and prescription drugs with Khat to heighten the effects or manage associated pain, and drug misuse by females and by health care professionals. The study also suggested that physicians and pharmacists fear counselling such people, probably with the risk of violence as a contributory factor.

## 1. Introduction

The misuse of medicinal products, both prescription and over-the-counter (OTC), is of increasing concern globally [1,2]. This may be related to their increased availability, inexpensive cost, and perceived safety [3,4]. Moreover, since 2010, stable or downward trends for traditional illicit drugs (heroin and cocaine) have been observed among major regions of consumption worldwide, but offset by an increase in synthetic and prescription drug use [5].

Access to medicinal products is subject to a variety of controls, which are decided at individual country level. The term ‘prescription only’ refers to medicinal products with public access controlled by prescription. Typically, in many countries, a prescription is a written instruction made by a medical doctor. Medicinal products available without a prescription can be referred to as ‘over the counter’ (OTC). Again, restrictions vary, with some medicines being limited for sale by a qualified pharmacist while others are available for purchase in retail outlets, such as grocery stores. There are several reports in the literature of prescription-only medications being sold without prescription in many countries and online [6,7]. Typically, in some countries of the Middle East and Africa, which are regions with political instability, war, and civil unrest, lack of social stability and safety for individuals are likely to be drivers for substance use. Although legislation does exist in these regions around medication supply, in many cases it is not strictly enforced. One can usually buy almost any medication, with the exception of narcotics and major tranquilizers, from community pharmacies [8].

Like in other countries in the region [8,9], the problem of dispensing medications that are liable for abuse without a valid prescription is a problem in Yemen [10]. Such drugs include analgesics, antibiotics, antipsychotic drugs, cardiovascular drugs, and others [8]. Medicinal drugs are not the only substances of abuse in Yemen. Khat chewing is legal, socially acceptable, and is a risk factor for the misuse of other drugs in the Yemeni community [11,12]. Khat (*Catha Edulis*) is a plant that contains an alkaloid, cathinone, an amphetamine-like stimulant that is believed to cause excitement, loss of appetite, and euphoria. It is commonly used as a social drug in the African horn and nearby countries like Yemen. The literature reports using prescription or OTC drugs either during (to potentiate its effects) [13] or after Khat chewing sessions, to minimize the unwanted CNS (Central Nervous System) and GIT (Gastrointestinal Tract) side effects [14]; The most frequently reported drugs in this regard were NSAIDs (Nonsteroidal Antiinflammatory Drugs), analgesics, sedatives, and tranquilizers [15]. In Aden in particular, the problem of drug abuse is increasing, which has been linked to an increase in violence and crime in the last three years [16].

Studies about drug abuse and misuse in Yemen are still scarce [17,18]. In one cross-sectional survey of 170 community pharmacists in Aden, 57.7% (*n* = 98) of respondents suspected drug misuse/abuse in users of their pharmacies and 83.8% (*n* = 142) noticed that it was increasing. Most commonly reported medications of abuse were Alprazolam, Ketoprofen, and Tramadol. The majority of suspects of prescription and non-prescription drug abuse (64.1%) were either chewing Khat or carrying it while buying the drug from the pharmacists [19]. Qualitative research complements quantitative work, so in order to help to build a wider picture of prescription and OTC drug abuse and misuse in Yemen, this study was implemented.

To the best of the authors’ knowledge, this is the first qualitative study that aimed to investigate users’ experiences regarding the misuse/abuse of OTC and prescription drugs in Yemen. It also aimed to understand the practice of mixing Khat with other drugs, including the sought effects and the users’ perceived consequences on health. Moreover, this study investigated the role (if any) that users thought health care providers could have in prescription and OTC drug misuse.

## 2. Materials and Methods

### 2.1. Study Sample and Recruitment

A purposive sample of customers suspected by the pharmacist on duty of misusing prescription/OTC medicines, attending nine community pharmacies in four districts of Aden, Yemen, was recruited. Recruitment was undertaken between April and September 2013. Pharmacists acted as gatekeepers to potential participants, introducing them to the researcher who took consent (see later). Community pharmacies were selected in different areas of Aden based on previous personal knowledge. in addition to snowball sampling. The community pharmacy was chosen as a safe and trusted recruitment point, where the researcher (EA) could approach suspected drug misusers and ask them to take part in the study. Pharmacies in areas of particular instability and violence had to be excluded for the personal safety of the researcher.

### 2.2. Consent and Data Collection

Due to safety and security issues as part of the political and social situation in Aden city, recruitment of subjects was considered difficult and risky. The safety of the researcher, a young woman, had to be considered in the design of this study. Thus, the following approach was taken: Interviews were conducted during the daytime. The researcher (EA) excluded those who were visibly carrying weapons, or those who appeared to be intoxicated or under withdrawal symptoms. Verbal consent to participate and audio-record the interviews was obtained. Interviews were conducted seated around a table in a quiet area inside the pharmacy, with the exception of pharmacies with no air-conditioner, where, for the purpose of comfort, interviews were conducted in the open air just outside the main entrance. Interviews were not conducted in private rooms, as the researcher was accompanied by 2 male relatives all the times. These ‘bodyguards’ were not present at the interviewing table and not close enough to be able to hear the conversation. In order to minimize potential bias that the presence of bodyguards could induce, participants were intentionally not informed of their presence. To further minimize bias, participants were assured of confidentiality and that the pharmacist would not be given information on what they said. The interviews were all conducted in Arabic. A topic guide directed the interviews (provided by the author upon request).

### 2.3. Data Management and Analysis

Data analysis was undertaken concurrently with data collection and continued until no new themes were being raised. Interviews were transcribed in Arabic and checked for accuracy by EA. Transcription and translation into English was also done by EA. For a sample of five transcripts, another researcher (MW) compared the translated English scripts with the original Arabic ones, back-translating them from English to Arabic. EA and MW then compared the original with the back-translated transcript and discussed consensus on accuracy. Thematic analysis was undertaken [20], guided by the research aims [21]. Salient quotes which illustrate the findings were identified and attributed to made-up initials. The precise steps employed in the qualitative analysis of interviews are summarized in Table 1. The transcriptions were independently analyzed by MW. EA and MW then compared their themes and subthemes and reached consensus through discussion to confirm the final results.

### 2.4. Ethical Considerations

The study was approved by The Higher Education Committee at The Faculty of Pharmacy/The University of Jordan, the IRB (Institutional Review Board) at The Jordan University Hospital (JUH; (#53/2013/IRB/J), and two Scientific Research Committees: At the School of Pharmacy and Deanship of Academic Research (DAR) at University of Jordan. There is no research ethics approval system in Yemen, so no local approvals could be sought. The work was done as an MSc project. All participants were assured of confidentiality and anonymity, and that they could contact the main researcher (EA) and withdraw from the study at any time prior to data analysis. Audio recordings were destroyed after analysis was complete.

## 3. Results

### 3.1. Study Sample

Data saturation was achieved after interviewing fifteen participants reported to be misusing OTC or prescription drugs. Interviews lasted in total between 30–50 min. Three female addicts declined participation because of social reasons. Most interviewees were male (*n* = 11) and between 21–30 years old (*n* = 9). Table 2 summarizes the demographic details of the interviewees.

### 3.2. Thematic Framework

Four main themes were identified during the interviews: (1) The pattern and methods of misuse; (2) the role of healthcare professionals in responding to misuse; (3) experience sought with drugs; and (4) awareness of problematic drug use. A number of subthemes emerged, such as different sources and street names of drugs, experimenting with different preparation methods and mixtures of OTC drugs with Khat or alcohol to enhance effects, in addition to using different methods (e.g., violence) to get drugs from doctors or pharmacists.

### 3.3. The Pattern and Methods of Misuse

#### 3.3.1. Frequency of Use

Drug misuse was very much related to Khat use; almost daily Khat consumption was accompanied with daily drug use. Three participants were an exception in this study, as they used drugs only, with no Khat.

In the case of psychoactive drugs, the majority were increasing the drug dose to get the same effect, while stable doses of analgesics (Ketoprofen, Ibuprofen, and Paracetamol) were noted.

#### 3.3.2. Preparation/Administration Methods

Three methods of mixing drugs with Khat were noted: (1) Grinding the drug tablets (e.g., BDZs (Benzodiazepines), Tramadol), and dissolving them in drinks; (2) grinding the drug tablets together with raw tobacco and placing them between the lower teeth gum and the inner lip membrane; and (3) chewing Ketoprofen with Khat and positioning on the painful tooth.

“Sometimes we don’t even know the name of the drug, anything we can try it with Khat, one day I attend a private Khat party we did at our friend’s home, and many tablets with different colors were inside a dish and anyone could take the tablet he liked and either drank it with water or coca cola, or chewed it together with Khat, tried it until we felt relaxed and happy.”

[Z, 25, M, undergraduate, Mix. Clonazepam/Khat]

“Sometimes I put one tablet under my tongue—speaking about Clonazepam- and another two tablets I dissolve with water or soft drinks during Khat session—stopped talking and asked the researcher if he can talk about new method other than Khat—recently there’s a new method called ‘Shamma*’ which is using raw tobacco leaves and drugs powder, usually we don’t know what a mix of drugs in this powder, put it all together between the cheek and gum, don’t chew it but keep it there and sucking as long as possible, even your lower lip getting a little bit swollen, the best thing of this way that no one can notice you as in Khat, there’s no ball in your cheek, there’s no smell like if you were taking alcohol, it’s hidden inside your inner lip with small quantity.”

[Z, 25, M, undergraduate, Mix. Clonazepam/Khat]

### 3.4. Role of Healthcare Professionals in Responding to Misuse

#### 3.4.1. Pharmacists

The study explored the participants’ experiences with pharmacists in terms of sale refusal and offer of help or advice. A common barrier to buying prescription medicines (without a prescription) was the pharmacist either refusing to sell the medicines or hiding them away from sight. The misusers would then make contact with dealers:

“I tried to buy it (Alprazolam) from the pharmacy but the pharmacist refused, now I don’t have to go the pharmacy as long as the drugs are available from drug dealers.”

[E, 24 M, undergraduate, Mix. Alprazolam/Khat]

However, others could get prescription-only medicines without a prescription as long as the pharmacist was known to them. Some misusers of OTC analgesics did not report any problems buying their OTC analgesics from pharmacies. OTC drugs, such as Paracetamol, Ketoprofen, and Solpadiene (*“codeine preparations with Paracetamol”*), were reported to be obtained either from the pharmacy, grocery shops, or supermarkets.

“No, the pharmacist doesn’t resist at all, he’s my friend. But if I don’t know the pharmacist I try to avoid asking this drug from him, he’ll refuse.”

(T, 35 M, Engineer, Mix. Tramadol/Khat)

Violence towards pharmacists was also reported. Some participants cited examples of weapons being used to force pharmacists to sell prescription medicines. Threatening pharmacists to steal drugs or money was also mentioned.

“Only before two years ago, my friend used his gun threatening the pharmacist to give us this drug, but not me I didn’t get involved in this.”

[HS, 24, M, undergraduate, Clonazepam]

#### 3.4.2. Physicians

Interviewees were asked if they had visited a physician for some medical reason, and whether the physician had noticed or suspected drug misuse. Most felt that their doctors never suspected their misuse of drugs, but focused only on their Khat consumption. Some interviewees did report that their doctor noticed their misuse of drugs and advised them directly or indirectly about the risks of these drugs. Participants thought that sometimes doctors do not like to ask about misuse or to get involved for safety reasons.

“Doctors are afraid to ask, there’s one doctor who asked my friend if he was taking any kind of drugs, but there was no action. The doctor can do nothing… Doctors are usually afraid to talk to families and even to us, because of weapons.”

[Z, 25, M, undergraduate, Mix. Clonazepam/Khat]

### 3.5. Experience Sought with Drugs

#### 3.5.1. Drugs of Misuse and Sources of Supply

Drug dealers and friends were the main sources of supply of drugs for about half of the participants, with the rest stating community pharmacies as their main source. OTC analgesics, such as Ketoprofen and Paracetamol, were reported to be easier to obtain, since they were available in supermarkets and grocery shops. Drug dealers were reported to be prevailing in low socioeconomic areas and to be well-known to people, especially in Khat-selling areas.

“Drug dealer in our street is well known years ago, and the police knows him as well, but every time he goes to prison, somehow he gets out, but everyone in the neighborhood knows him.”

[SH, 24, M, undergraduate, Clonazepam]

“I can get the drugs from the same place I get Khat, but only for those who know how, you can find a Somali shoe cleaner, there’s a secret code about the color you want on your shoes, if you say blue, then he understands that you want Viagra, if you say red, that means you want Tramadol.”

[SS, 40, M, employed, Alprazolam]

#### 3.5.2. Street Names of Drugs

“Al-Qathafi” was the most famous street name for Alprazolam, and there was one street name in common for any drug being abused, “Qara’a” كرع, which is a general local term given to any pill mixed with Khat. Clonazepam had more than one street name; none of the interviewees knew its medical name, so they called it “Abo-Saleeb” or “Roch”.

“It has a crosslike shape on the tablet ‘Saleeb’ (drawing it on his hand) so we call it ‘abu-Saleeb’, or Roch because it’s written on its cover, it’s in 2 mg and 1 mg, isn’t it?”

[Z, 24 M, undergraduate, Mix. Clonazepam/Khat]

On the other hand, Tramadol was simply called “Qara’a”كرع. Some called it “Al-Ahmar” (also known as “the red one”); others called it simply “Tramadol”.

“I don’t know what its name, my friend bring it to me (showing the researcher the tablets) but when I need it I tell her give me that red tablet.”

[R, 32 F, Housewife, Tramadol]

#### 3.5.3. First Experiences with Drugs

Almost all of the participants in this study tried prescription/OTC drugs for the first time with their friends.

“My friends told me about “Abo-Saleeb” (Clonazepam) during our Khat session especially on that day, they noticed me having serious problems in my home, just for fun and to change my mood.”

[AA, 24 M, undergraduate, Mix. Clonazepam/Khat]

“The beginning was just a curiosity, I liked to try it because of a big problem happened to me at that time, it was my escapism, and even sometimes Tramadol doesn’t work enough with me as it did at the beginning so I added Alprazolam just the lowest dose 0.25 mg.”

[M, 35 F, Physician, Tramadol]

#### 3.5.4. Description of Drug Effect

Drug effects sought by participants varied, based on the drug and the dose used and whether they mixed it with Khat or not. However, all who were combining Alprazolam with Khat had almost the same description for the experienced effect, as illustrated by ES:

“When I take it (Alprazolam) with Khat, I feel good and sometimes I see one person as two (laughing). I forget everything at the same time feel powerful and everything is under control.”

[ES, 20 M, undergraduate, Mix. Alprazolam/Khat]

“It (the effect) depends on the dose of the tablet (Alprazolam). The taste of Khat gets better. I mean its effect gets better, my whole body feels relaxed and can’t think of any problem. But sometimes with high dose I lose the ability to walk.”

[N, 24 M, a pharmacist, Mix. Alprazolam/Khat]

As for Tramadol, the experienced effect depended on whether it was mixed with Khat or not, and the motivation was different. Those who were taking it without Khat actually sought pain relief, and then discovered its relaxing effect.

“Once I take this tablet (Tramadol), I can do my activities at home very well in a perfect way without being nervous, and don’t feel my back pain at all.”

[R, 32, F, Housewife, Tramadol]

On the other hand, some Khat chewers took Tramadol in combination with Khat for mental-altering effects, as in the following case:

“Taking it (Tramadol) with Khat works like magic for me, after 15 minutes it makes me relaxed and completely calm.”

[T, 35, M, Engineer, Mix. Tramadol/Khat]

Clonazepam was felt to have the most potent effect when mixed with Khat, and its withdrawal symptoms, such as aggression, were obvious the following day.

“My best friend broke my lower jaw, suddenly next day came and hit me for no reason.”

[Z, 25, M, Unemployed, Mix. Clonazepam/Khat]

Regarding OTC drug misuse (Chlorpheniramine and Ketoprofen), two interviewees had different motivations and reasons to use it with Khat:

“I can’t have Khat without Ketoprofen, it relieves my gum and teeth pain during long Khat sessions. Without placing Ketoprofen in the place where I’m chewing my Khat –left or right cheek- I can’t keep long Khat session with friends.”

[NT, 22, M, undergraduate, Mix. Ketoprofen/Khat]

“While chewing Khat in a room with cold air-conditioner, I can’t breathe well feeling nasal congestion, Histop [brand name of Chlopheniramine], relieves my nasal congestion so I breathe easily and enjoy a long Khat session with friends, somehow it keeps me calm also.”

[AM, 55 M, retired, Mix. Chlorpheneramine/Khat]

#### 3.5.5. Reported Side Effects and Withdrawal Symptoms

The majority of interviewees did not consider that they had experienced any unwanted effects, while some reported hypotension, vomiting, dizziness, loss of consciousness, and renal colic (reported by those using Ketoprofen and Khat). One participant reported falling down due to severe hypotension, and required I.V fluids. Another participant described an overdose in three of his friends after a high dose of Clonazepam.

“Three of my friends had their hearts stop pumping, we took them to the hospital and only with CPR they woke up and survived.”

[Z, 25, M, undergraduate, Mix. Clonazepam/Khat]

He also described aggressiveness felt the following day:

“The problem is that after Khat and even the next day I’m not myself, for no reason I’m relaxed but aggressive at the same time, feel like I’m the king of the world, anyone who try to say anything to me I’m ready to kill him, feeling very strong, the same with my friends, (pointing his finger to his lower jaw asking the researcher) do you see this deviation in my face? My best friend broke my lower jaw, suddenly next day came and hit me for no reason.”

### 3.6. Awareness of Problematic Drug Use

#### 3.6.1. Reasons to Continue Drug Misuse

The main reasons for continuing to misuse Alprazolam, Tramadol, and Clonazepam were similar (e.g., calmness, joyful time with friends during Khat sessions, escapism from pressures and daily problems).

“Relaxes me during fishing at late night especially with Khat, it makes me awake and relaxed at the same time with no stress at all, don’t feel sea sickness or panic.”

[F, 28, M, fisherman, Mix. Alprazolam/Khat]

#### 3.6.2. Contemplation of There Being a Problem

Interviewees differed in their contemplation of whether they had a “drug problem”. This was related to the type of drug used and individual characteristics. The researcher was using the terminologies of the question in Arabic language translated as: *Do you feel that, by using the pills this way, you are “abusing” these drugs or you are so “addicted” to them that you cannot stop?*

The majority of the participants avoided describing it as an “addiction”; some of them preferred to call it “abuse” but not “addiction”, while others did not consider it is a serious problem.

“I may consider myself as an abuser…[but] we can stop it anytime, we’re not addicts, and it’s just for mood enhancement with friends. Especially during this difficult time, I have no problem with drugs it’s just like Khat.”

[24, M, undergraduate, Clonazepam]

“I think I get used to it, but not at a level of addiction, I tried to stop it, but Khat is not good without it, I just use it to make me feel good with Khat only.”

[E, 24 M, undergraduate, Mix. Alprazolam/Khat]

“No it’s not a problem or an addiction, it’s more likely to be a psychological problem, you can call it abuse if you want to.”

[MM, 35 F, Physician, Tramadol]

On the other hand, there were those who were afraid of addiction and had been trying so hard to stop use or find an alternative.

“I can’t deny it, I feel I’m in trouble with this drug (Tramadol) and maybe an addiction, recently I’ve tried to stop it but didn’t know how to do so.”

[T, 35 M, Engineer, Mix. Tramadol/Khat]

“I hope if there’s an alternative (Ketoprofen) I want to stop it, yes I feel I have problem with it and the dentist told me it may destroy my teeth with Khat, and I have kidney pain also, but I can’t chew Khat without Ketoprofen, my gum and teeth pain is severe without it.”

[NT, 22 M, undergraduate, Mix. Ketoprofen /Khat]

“No, I don’t want to stop Alprazolam at all, even if I’m an addict, I’m afraid to come back to alcohol again. Restyl (brand name of Alprazolam) is ok with me, and better than getting addicted to alcohol.”

[SS, 40 M, employed, Mix. Alprazolam/Khat]

#### 3.6.3. Drug Seeking Behavior

Reported drug-seeking behavior differed depending on the type of drug. OTC analgesics (e.g., Ketoprofen, Ibuprofen) are readily available in pharmacies, supermarkets, and grocery shops with affordable costs and thus, drug-seeking behavior was not found to be problematic. On the other hand, for Tramadol, some of interviewees had to lie to get money or to steal from their families to obtain it. Few participants reported using their guns, and threatening pharmacists to steal drugs or money.

“Only before two years ago, my friend used his gun threatening the pharmacist to give us this drug, but not me I didn’t get involved in this.”

[HS, 24, M, undergraduate, Clonazepam]

## 4. Discussion

### Summary of Main Findings

This study was the first to provide in-depth qualitative data on the experience of prescription and OTC drug misusers in Aden, Yemen. It further explains the findings reported in the quantitative part of the study, which reported the mixing of drugs with Khat [19]. This paper describes methods of preparation, mixing and administering prescription/OTC medicines with Khat, and the types of prescription and OTC drugs misused by participants, many of whom also used Khat. The only substance mentioned previously in the literature to be mixed with Khat was tobacco [11], and this was attributed to the claimed analgesic effects of tobacco, which may have masked the pain of Khat chewing [22]. This study showed, for the first time, OTC analgesics such as Ketoprofen to be used to treat mouth pain from Khat chewing. Further different effects sought and obtained by mixing prescription/OTC medicines with Khat are also explained. Unpleasant physical withdrawal effects, aggressiveness, and psychological symptoms were reported by participants. This suggests that any interventions to address Khat use need to be cognizant of the possibility of polydrug use and dependence.

The study also shed light on violence in pharmacy practice, especially in areas of armed conflict. This has not previously been reported in the literature. This was reflected in the reported use of weapons by pharmacy customers and threatening of pharmacists and/or doctors. Carrying traditional weapons, such as Jambia (dagger), is part of the traditional Yemeni costume. Moreover, the spread of guns and modern weapons has been increasingly prevalent in the last few years [23]. Similar violence and aggression among drug misusers has been reported in other studies in different parts of the world [24,25]. This violence was also reflected in the perception that doctors and pharmacists were afraid to challenge or counsel individuals suspected of drug misuse. Manchikanti et al.’s research [26] suggests that there is a lack of training on how to deal with such violent cases. Also, it is believed to be very unlikely for interventions or health advice to be offered in a violent atmosphere [25].

There have currently been no studies in Yemen that look at drug abuse among health care professionals. However, this risk was evident in this study by the inclusion of two participants who were a doctor and a pharmacist respectively. Previous studies in other countries have attributed doctor’s misuse of medicines to ease of access to drugs, in addition t[o overconfidence and the stressful style of their job [27,28]. Similar reasons have also been reported for misuse of drugs by pharmacists and pharmacy students [29,30].

The combination of sedatives with stimulants was commonly reported during Khat sessions amongst participants. This reflects similar polydrug use findings reported in Ethiopia and Kenya, where Khat chewing was frequently mixed with alcohol and benzodiazepine use [31,32].

The mixing of Khat, which has stimulant properties, with sedatives, including alcohol and benzodiazepines could heighten risk [33], as the user may find the sedation of the benzodiazepines is counteracted by the Khat. Additionally, the risks of combining two sedatives such as alcohol and benzodiazepines may not be felt. This might result in the person consuming sedatives beyond his limits, leading to sedative overdose [34].

Risky behaviors by drug misusers in Yemen were also described; for example, the ignorance of the contents of drugs prior to consumption in combination with Khat. There were reports where the name of the drug taken was not known, suggesting a likely lack of access to information on risk and potential harms. Similarly, a lack of acknowledgement of addiction despite the reporting of addictive behaviors was evident amongst participants. Most interviewees in this study denied having a problem with drugs. Addiction denial is a recognized behavior at some stages of dependence [35]. This denial is reported to be reduced when addicts are following a rehabilitation program [36], presumably because those who enter rehabilitation have contemplated their problem and have motivation to address it. It may, however, be that the context of this study prevented disclosure of addiction. This is a very sensitive issue, and participants may have felt that disclosure might impede their ability to buy prescription or OTC medicines in the pharmacy where they were recruited. None of the participants in this study reported being in any kind of treatment or rehabilitation program. There is only one rehabilitation program in Yemen, which was established in 2009 in Sana’a [37]. This study suggests there is potential for greater prevention and harm reduction advice to be given to people at risk of drug misuse in Yemen. Further work is needed to establish whether pharmacists and doctors could play a role in the provision of such advice, and to map the potential for such intervention development and evaluation [38].

This study also highlights the social nature of prescription and OTC drug misuse amongst participants. It showed that being with friends in a Khat session plays an important role as a motivation factor for misusing other (prescription or non-prescription) drugs, whether to enhance the euphoric effect of the Khat [30], or to mask Khat chewing problems such as mouth pain, as reported in this study and other studies [33,34]. This suggests that interventions to discourage prescription and OTC drug misuse in Yemen need to take the context of Khat sessions into account and provide information and support appropriate for this social setting. Harm reduction advice could include discouraging the removal of medicines from their packaging to allow identification, accompanied by the provision of clear information on risks and harms.

## 5. Strengths and Limitations of the Study

This study is the first in Yemen to highlight prescription and OTC medicine misuse from the users’ perspective. It has provided novel descriptive information on prescription and OTC medicine consumption, mixed with the more traditional use of Khat to either heighten the psychoactive experience or to combat pain from Khat chewing. It also describes violence as a barrier to effective pharmacy practice, something not previously noted in the literature. The sample recruited in this study varied in age, gender, level of education, and occupation. It included elderly people, females, and health care professionals. These groups can be difficult to recruit because of the sensitivity of the issue in Yemeni culture, so their representation is a strength point of the study. Moreover, carrying out such a study about a stigmatizing issue in a war zone like Yemen was a great risk that would add to the strength and novelty of this study.

The main limitation of the study was the challenges in recruitment, which was slow and had to rely on convenience and snowball sampling due to the difficult-to-reach nature of the sampling frame and the stigma associated with drug misuse. Many people were hesitant to take part, despite the assurance of confidentiality and anonymity of data. Although every effort was made not to mention the faculty of pharmacy at which the researcher was based, they knew the researcher was a pharmacist and therefore the risk of social desirability bias cannot be excluded. The unsecure conditions in Aden at the time of carrying out the study (2014) meant the researcher had to be accompanied by two male relatives, which may be criticized as having the potential to impede the free speaking of participants. The presence of these ‘bodyguards’ was not explicitly made known to participants. Similarly, data were not collected in a private room, but rather in a quiet corner of the pharmacy. There also has to be caution in assuming the implications of such practice on research collected in a Western setting would translate to the Yemeni context. The researcher was a lone female and, in the Yemeni context, the closed-door environment of a private room would not be acceptable.

Finally, the study was conducted in Aden, the biggest urban center in the south of Yemen. It was qualitative in nature, seeking to understand experiences and not to quantify them. Therefore, the results are not generalizable across Yemen.

## 6. Conclusions

This study qualitatively describes the experience of misusers and abusers of prescription and non-prescription drugs in Yemen. Most users do not contemplate their behavior as an addiction problem. Mixing Khat with drugs is common and takes place for different reasons. The lifestyle, culture, and political situation are all complicating factors for health care interventions and evaluation.

## Figures and Tables

**Table 1:** The precise steps employed in the analysis of interviews with suspected drug abusers in Aden [21].

**Step 1**	For each interview, transcripts were read, re-read and summary notes were made and grouped, under potential thematic categories.
**Step 2**	As analysis progressed, the points of interest were reorganized and categorized under different themes, which were often renamed when a more appropriate title emerged.
**Step 3**	Thematic analysis was carried out as themes were encountered on each tape, with particular attention being paid to any cases which agreed with existing themes (Pope et al., 2000). Negative cases or alternative explanations were also sought, in order to confirm the themes identified from each separate interview.
**Step 4**	Attention was also paid to whether themes were common across the interviews or were specific to one interview, in order to note any patterns emerging from the data. Any new themes that emerged were also noted and some themes were grouped together or recategorized to better fit the data.
**Step 5**	This resulted in the production of a more definitive list of emergent themes, which were felt to reflect the data produced from the interviews.
**Step 6**	The transcriptions were independently analyzed by another researcher (MW) and, when both researchers had reached consensus, these analytic themes were confirmed and translated into an account of the shared experience among the participating pharmacists.

**Table 2:** Case numbers and demographic characteristics of suspected prescription and over-the-counter (OTC) drug abusers in Aden. (*n* = 15).

Items	Frequency (%)
**Marital status**	
1. Married	4 (26.6)
2. Single	6 (40.0)
3. Divorced	5 (33.3)
**Age**	
1. 21–30	9 (60.0)
2. 31–40	4 (26.6)
3. 40–65	2 (13.3)
**Gender**	
Male	11 (73.3)
Female	4 (26.6)
**Drug of abuse**	
Tramadol	4 (26.6), 2 mixed with Khat
Alprazolam	5 (33.3), 4 mixed with Khat
Clonazepam	2 (13.3), both mixed with Khat
Ketoprofen	2 (13.3), 1 mixed with Khat
Chlorpheniramine	1 (6.7), mixed with Khat
Mix (Ibuprofen+Paracetamol)	1 (6.7)
**Duration of drug abuse**	
<1 year	1 (6.7)
1–2 years	2 (13.3)
2–5 years	8 (53.3)
>5 years	4 (26.6)
**Education level**	
1. Primary/preparatory	5 (33.3)
2. Secondary undergraduate	4 (26.6)
3. Secondary/university	6 (40.0)
**Occupation**	
Employed	6 (40)
Unemployed	6 (40)
Retired	2 (13.3)
Private business	1 (6.7)
